# 2-Tosyl-2,3,3a,4,9,9a-hexa­hydro-1*H*-benzo[*f*]isoindol-1-one

**DOI:** 10.1107/S1600536813011045

**Published:** 2013-05-18

**Authors:** Min Wu, Yi-Min Hu

**Affiliations:** a School of Chemistry and Materials Science, Anhui Normal University, Wuhu, Anhui 241000, People’s Republic of China

## Abstract

The title compound, C_19_H_19_NO_3_S, was produced by the self-reaction of *N*-cinnamyl-*N*-tosyl­acryl­amide in the presence of palladium(II) acetate *via* an intra­molecular C—C coupling reaction and C—H activation. There are two chiral C atoms in the mol­ecule, but the crystal is a racemic system due to a lack of chiral separation. The five-membered ring is twisted about the methyl­ene–methane bond, and the cyclo­hexa-1,4-diene ring has a boat conformation. The dihedral angle between the benzene rings is 88.27 (14)°, indicating an almost orthogonal relationship and an approximate L-shape for the mol­ecule. In the crystal, the presence of C—H⋯π inter­actions leads to inversion dimers.

## Related literature

For palladium-catalysed inter­molecular and intra­molecular reactions, see: Zhao *et al.* (2012 ▶) and for palladium-catalysed coupling reactions, see: Meng *et al.* (2011 ▶); Hu *et al.* (2011 ▶). They have made a wide variety active pharmaceutical ingredients and complex organic mol­ecules economically accessible, see: Hu *et al.* (2009 ▶, 2010 ▶). For the physiological activity of benzo[*f*]isoindol-1-one derivatives, see: Pitchumani & Vijaikumar (2010 ▶).
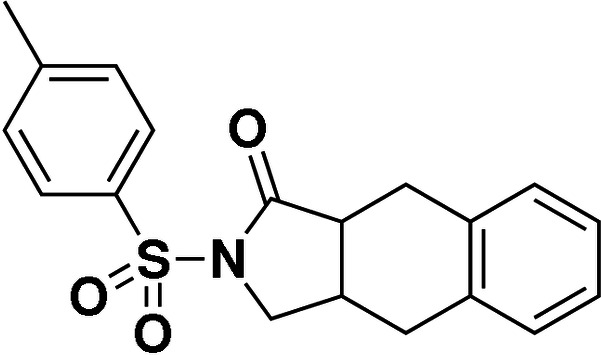


## Experimental

### 

#### Crystal data

C_19_H_19_NO_3_S
*M* *_r_* = 341.41
Triclinic, 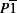

*a* = 6.4389 (8) Å
*b* = 8.4336 (11) Å
*c* = 15.4958 (12) Å
α = 89.312 (2)°
β = 87.395 (3)°
γ = 81.224 (2)°
*V* = 830.75 (16) Å^3^
*Z* = 2
Mo *K*α radiation
μ = 0.21 mm^−1^
*T* = 291 K
0.28 × 0.24 × 0.22 mm

#### Data collection

Bruker SMART APEX CCD diffractometer
Absorption correction: multi-scan (*SADABS*; Bruker, 2000 ▶) *T*_min_ = 0.943, *T*_max_ = 0.955
7242 measured reflections
3724 independent reflections
1902 reflections with *I* > 2σ(*I*)
*R*_int_ = 0.041

#### Refinement

*R*[*F* ^2^ > 2σ(*F* ^2^)] = 0.055
*wR*(*F* ^2^) = 0.140
*S* = 1.00
3724 reflections
218 parameters
1 restraint
H-atom parameters constrained
Δρ_max_ = 0.19 e Å^−3^
Δρ_min_ = −0.27 e Å^−3^

### 

Data collection: *SMART* (Bruker, 2000 ▶); cell refinement: *SAINT* (Bruker, 2000 ▶); data reduction: *SAINT*; program(s) used to solve structure: *SHELXTL* (Sheldrick, 2008) ▶; program(s) used to refine structure: *SHELXTL*; molecular graphics: *SHELXTL*; software used to prepare material for publication: *SHELXTL*.

## Supplementary Material

Crystal structure: contains datablock(s) global, I. DOI: 10.1107/S1600536813011045/bv2220sup1.cif

Structure factors: contains datablock(s) I. DOI: 10.1107/S1600536813011045/bv2220Isup2.hkl

Supplementary material file. DOI: 10.1107/S1600536813011045/bv2220Isup3.cml

Additional supplementary materials:  crystallographic information; 3D view; checkCIF report

## Figures and Tables

**Table 1:** Hydrogen-bond geometry (Å, °) *Cg*1 is the centroid of the C1–C6 ring.

*D*—H⋯*A*	*D*—H	H⋯*A*	*D*⋯*A*	*D*—H⋯*A*
C10—H10*A*⋯*Cg*1^i^	0.97	2.63	3.555 (3)	159

Symmetry code: (i) 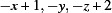.

**Fig. 1.**
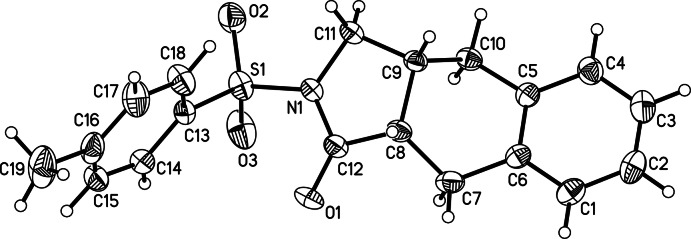
A view of the title compound showing the atom-numbering scheme and displacement ellipsoids drawn at 30% probability level.

**Fig. 2.**
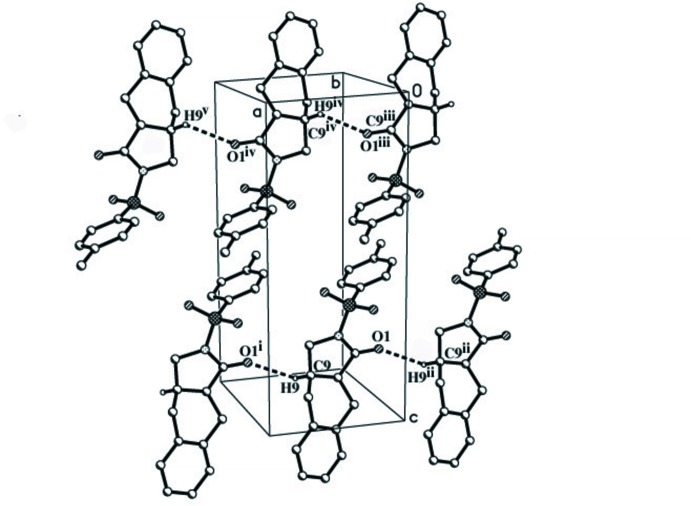
A view of the forming a 1-D chain along the a axis. (i: 1 + x, y, z; ii: -1 + x,y,z; iii:-x,1 - y,1 - z; iv: 1 - x,1 - y,1 - z; v: 2 - x,1 - y,1 - z)

**Fig. 3.**
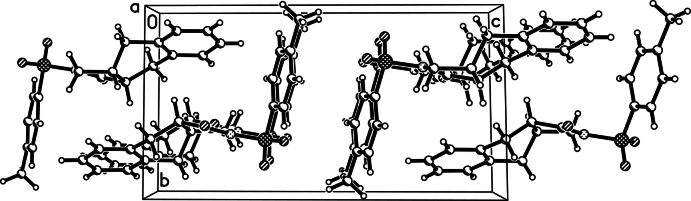
A view of the cell packing.

**Fig. 4.**
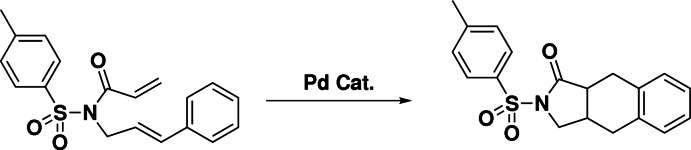
The formation of the title compound.

## supplementary crystallographic information

### Comment

Palladium-catalyzed intermolecular and intramolecular reactions have become an important tool of modern organic synthesis chemistry(Zhao *et al.*, 2012). They have made a wide variety of active pharmaceutical ingredients and complex organic molecules economically accessible(Hu *et al.*, 2009; Hu *et al.*, 2010). The benzo[*f*]isoindol-1-one derivatives, which have physiological activity, are effective intermediates in the preparation of many complex natural products(Pitchumani *et al.*, 2010). We have reported some novel palladium-catalyzed coupling reactions of aryl halides with the olefins and dienes(Meng *et al.*, 2011; Hu *et al.*, 2011). The self-reaction of *N*-cinnamyl-*N*-tosylacrylamide, in the presence of palladium(II) acetate and triphenylphosphine, in DMF at 393 K for 16 h, gave the unexpected title product.

The crystal structure data of molecule (I), C_19_H_19_NO_3_S, reveals that all the bond lengths and angles have normal values. An asymmetric unit is composed of one title compound molecule. The title compound molecule contains two phenyl ring, one five-member carbon ring, and one six-member carbon ring. All the rings are not coplanar (figure 1). In the molecule (I) there are two chiral carbon atoms, C8 and C9, but the crystal is a racemic system due to lacking of the chiral separation. The five-membered ring is twisted about the methylene–methane bond, and the cyclohexa-1,4-diene ring has a boat conformation. The dihedral angle between the benzene rings is 88.27 (14)°, indicating an almost orthogonal relationship and an approximate L-shape for the molecule. In the crystal, the presence of C—H···π interactions leads to inversion dimers. The molecules with *R*, S(C8, C9) conformation form a 1-D chain through weak H9···O_1_^i^(i: 1 + *x*, *y*,*z*) interactions, so do molecules with S, *R*(C8^iii^, C9^iii^) conformation (H9^iii^···O_1_^iv^ (iii: -*x*, 1 - *y*,1 - *z*; iv: 1 - *x*,1 - *y*,1 - *z*). Two chains are parallel with each other along the *a* axis(Fig. 2).

### Experimental

An oven-dried Schlenk flask was evacuated, filled with nitrogen, and then charged with *N*-cinnamyl-*N*-tosylacrylamide (3.41 g, 10 mmol), tributylamine (3 ml), PPh_3_ (52.5 mg, 0.2 mmol), Pd(OAc)_2_ (24 mg, 0.1 mol), and DMF (10 ml) to give a yellow solution. The reaction mixture was heated at 393 K with stirring. The reaction mixture was cooled to room temperature after 16 h and the resultant yellow-orange mixture was diluted with Et_2_O (10 ml). The mixture was washed with H_2_O (15 ml) and the aqueous layer was extracted with Et_2_O (20 ml). The combined organic layers were dried (MgSO_4_), filtered, and concentrated *in vacuo*. The crude material was purified by flash column chromatography on silica gel (petroleum ester:EtOAc = 7:1) and recrystalized from EtOAc, yield 3.11 g (91%). Colorless crystals suitable for X-ray diffraction were obtained by recrystallization from a solution of the title compound from ethyl acetate over a period of one week.

### Refinement

H atoms were positioned geometrically and refined using a riding model with C—H = 0.93–0.97 Å, and with *U*_iso_(H) = 1.2 times *U*_eq_(C).

### Crystal data

**Table:**

C_19_H_19_NO_3_S	*Z* = 2
*M**_r_* = 341.41	*F*(000) = 360
Triclinic, *P*1	*D*_x_ = 1.365 Mg m^−^^3^
Hall symbol: -P 1	Mo *K*α radiation, λ = 0.71073 Å
*a* = 6.4389 (8) Å	Cell parameters from 3121 reflections
*b* = 8.4336 (11) Å	θ = 2.1–23.6°
*c* = 15.4958 (12) Å	µ = 0.21 mm^−^^1^
α = 89.312 (2)°	*T* = 291 K
β = 87.395 (3)°	Block, colorless
γ = 81.224 (2)°	0.28 × 0.24 × 0.22 mm
*V* = 830.75 (16) Å^3^	

### Data collection

**Table:**

Bruker SMART APEX CCD diffractometer	3724 independent reflections
Radiation source: sealed tube	1902 reflections with *I* > 2σ(*I*)
Graphite monochromator	*R*_int_ = 0.041
phi and ω scans	θ_max_ = 27.5°, θ_min_ = 1.3°
Absorption correction: multi-scan (*SADABS*; Bruker, 2000)	*h* = −8→8
*T*_min_ = 0.943, *T*_max_ = 0.955	*k* = −10→10
7242 measured reflections	*l* = −20→18

### Refinement

**Table:**

Refinement on *F*^2^	Primary atom site location: structure-invariant direct methods
Least-squares matrix: full	Secondary atom site location: difference Fourier map
*R*[*F*^2^ > 2σ(*F*^2^)] = 0.055	Hydrogen site location: inferred from neighbouring sites
*wR*(*F*^2^) = 0.140	H-atom parameters constrained
*S* = 1.00	*w* = 1/[σ^2^(*F*_o_^2^) + (0.0538*P*)^2^ + 0.0132*P*] where *P* = (*F*_o_^2^ + 2*F*_c_^2^)/3
3724 reflections	(Δ/σ)_max_ = 0.002
218 parameters	Δρ_max_ = 0.19 e Å^−^^3^
1 restraint	Δρ_min_ = −0.27 e Å^−^^3^

### Special details

**Table:**

Geometry. All e.s.d.'s (except the e.s.d. in the dihedral angle between two l.s. planes) are estimated using the full covariance matrix. The cell e.s.d.'s are taken into account individually in the estimation of e.s.d.'s in distances, angles and torsion angles; correlations between e.s.d.'s in cell parameters are only used when they are defined by crystal symmetry. An approximate (isotropic) treatment of cell e.s.d.'s is used for estimating e.s.d.'s involving l.s. planes.
Refinement. Refinement of *F*^2^ against ALL reflections. The weighted *R*-factor *wR* and goodness of fit *S* are based on *F*^2^, conventional *R*-factors *R* are based on *F*, with *F* set to zero for negative *F*^2^. The threshold expression of *F*^2^ > σ(*F*^2^) is used only for calculating *R*-factors(gt) *etc*. and is not relevant to the choice of reflections for refinement. *R*-factors based on *F*^2^ are statistically about twice as large as those based on *F*, and *R*- factors based on ALL data will be even larger.

### Fractional atomic coordinates and isotropic or equivalent isotropic displacement parameters (Å^2^)

**Table:**

	*x*	*y*	*z*	*U*_iso_*/*U*_eq_	
C1	0.3560 (5)	0.2477 (3)	1.14379 (19)	0.0529 (8)	
H1	0.2229	0.2911	1.1658	0.063*	
C2	0.5104 (6)	0.1853 (4)	1.1995 (2)	0.0632 (9)	
H2	0.4793	0.1846	1.2587	0.076*	
C3	0.7085 (6)	0.1248 (4)	1.1681 (2)	0.0632 (9)	
H3	0.8121	0.0837	1.2058	0.076*	
C4	0.7530 (5)	0.1253 (3)	1.0804 (2)	0.0516 (8)	
H4	0.8885	0.0863	1.0593	0.062*	
C5	0.6001 (4)	0.1827 (3)	1.02266 (18)	0.0402 (7)	
C6	0.3994 (4)	0.2456 (3)	1.05538 (18)	0.0420 (7)	
C7	0.2351 (4)	0.3035 (4)	0.99167 (19)	0.0509 (8)	
H7A	0.1217	0.3744	1.0211	0.061*	
H7B	0.1771	0.2121	0.9711	0.061*	
C8	0.3179 (4)	0.3927 (3)	0.91382 (17)	0.0402 (7)	
H8	0.2959	0.5078	0.9264	0.048*	
C9	0.5518 (4)	0.3398 (3)	0.88727 (16)	0.0383 (7)	
H9	0.6331	0.4209	0.9067	0.046*	
C10	0.6378 (4)	0.1798 (3)	0.92661 (17)	0.0421 (7)	
H10A	0.5702	0.0963	0.9024	0.050*	
H10B	0.7876	0.1552	0.9127	0.050*	
C11	0.5606 (4)	0.3383 (4)	0.78857 (18)	0.0474 (8)	
H11A	0.6051	0.4355	0.7652	0.057*	
H11B	0.6567	0.2464	0.7666	0.057*	
C12	0.1997 (5)	0.3657 (4)	0.83510 (19)	0.0478 (7)	
C13	0.1097 (4)	0.4760 (3)	0.64007 (17)	0.0424 (7)	
C14	−0.0883 (4)	0.4722 (4)	0.61141 (18)	0.0510 (8)	
H14	−0.1412	0.3758	0.6087	0.061*	
C15	−0.2072 (5)	0.6144 (5)	0.58664 (19)	0.0614 (9)	
H15	−0.3399	0.6120	0.5658	0.074*	
C16	−0.1359 (6)	0.7594 (4)	0.59177 (19)	0.0621 (9)	
C17	0.0634 (6)	0.7592 (4)	0.6211 (2)	0.0703 (10)	
H17	0.1151	0.8558	0.6252	0.084*	
C18	0.1867 (5)	0.6194 (4)	0.6445 (2)	0.0631 (9)	
H18	0.3215	0.6214	0.6632	0.076*	
C19	−0.2678 (6)	0.9147 (5)	0.5653 (2)	0.0981 (14)	
H19A	−0.2730	0.9919	0.6106	0.147*	
H19B	−0.4078	0.8957	0.5549	0.147*	
H19C	−0.2064	0.9551	0.5135	0.147*	
N1	0.3425 (3)	0.3281 (3)	0.76626 (14)	0.0429 (6)	
O1	0.0116 (3)	0.3754 (3)	0.83041 (14)	0.0784 (8)	
O2	0.4664 (3)	0.2878 (3)	0.61566 (13)	0.0654 (6)	
O3	0.1562 (4)	0.1696 (2)	0.66729 (15)	0.0769 (7)	
S1	0.27381 (13)	0.29881 (9)	0.66632 (5)	0.0514 (3)	

### Atomic displacement parameters (Å^2^)

**Table:**

	*U*^11^	*U*^22^	*U*^33^	*U*^12^	*U*^13^	*U*^23^
C1	0.062 (2)	0.0443 (18)	0.051 (2)	−0.0084 (16)	0.0091 (17)	−0.0015 (15)
C2	0.088 (3)	0.062 (2)	0.0405 (19)	−0.015 (2)	−0.0038 (19)	−0.0014 (16)
C3	0.073 (3)	0.062 (2)	0.056 (2)	−0.012 (2)	−0.0174 (19)	0.0071 (17)
C4	0.0528 (19)	0.0468 (18)	0.056 (2)	−0.0073 (15)	−0.0084 (16)	0.0053 (15)
C5	0.0443 (17)	0.0313 (15)	0.0456 (18)	−0.0079 (13)	−0.0024 (14)	0.0049 (12)
C6	0.0468 (18)	0.0358 (16)	0.0434 (18)	−0.0082 (14)	0.0033 (14)	0.0000 (13)
C7	0.0381 (17)	0.0528 (19)	0.060 (2)	−0.0048 (14)	0.0082 (15)	0.0021 (15)
C8	0.0320 (16)	0.0358 (16)	0.0514 (18)	−0.0013 (12)	0.0003 (13)	0.0006 (13)
C9	0.0311 (15)	0.0395 (16)	0.0451 (17)	−0.0082 (12)	−0.0022 (12)	0.0026 (12)
C10	0.0351 (16)	0.0374 (16)	0.0523 (19)	−0.0018 (13)	0.0004 (13)	−0.0018 (13)
C11	0.0303 (16)	0.058 (2)	0.0524 (19)	−0.0017 (14)	−0.0056 (13)	0.0079 (14)
C12	0.0337 (17)	0.055 (2)	0.054 (2)	−0.0039 (14)	−0.0043 (15)	0.0103 (15)
C13	0.0415 (17)	0.0445 (17)	0.0409 (17)	−0.0038 (14)	−0.0073 (13)	0.0063 (13)
C14	0.0422 (18)	0.065 (2)	0.0459 (19)	−0.0080 (16)	−0.0033 (15)	0.0028 (15)
C15	0.0412 (19)	0.089 (3)	0.049 (2)	0.0066 (19)	−0.0047 (15)	0.0037 (19)
C16	0.068 (2)	0.069 (2)	0.0385 (19)	0.0218 (19)	0.0013 (17)	0.0017 (16)
C17	0.089 (3)	0.050 (2)	0.070 (2)	−0.001 (2)	−0.020 (2)	0.0058 (18)
C18	0.057 (2)	0.055 (2)	0.078 (3)	−0.0072 (18)	−0.0216 (18)	0.0065 (18)
C19	0.115 (3)	0.087 (3)	0.073 (3)	0.046 (3)	−0.009 (2)	0.013 (2)
N1	0.0325 (13)	0.0520 (15)	0.0437 (15)	−0.0036 (11)	−0.0070 (11)	0.0052 (11)
O1	0.0266 (12)	0.135 (2)	0.0729 (17)	−0.0107 (13)	−0.0060 (11)	0.0115 (15)
O2	0.0576 (12)	0.0761 (16)	0.0547 (14)	0.0166 (12)	−0.0041 (9)	−0.0101 (11)
O3	0.0937 (18)	0.0507 (14)	0.0942 (18)	−0.0258 (13)	−0.0428 (14)	0.0088 (12)
S1	0.0530 (5)	0.0458 (5)	0.0553 (5)	−0.0035 (4)	−0.0151 (4)	−0.0002 (4)

### Geometric parameters (Å, º)

**Table:**

C1—C2	1.385 (4)	C11—N1	1.477 (3)
C1—C6	1.385 (4)	C11—H11A	0.9700
C1—H1	0.9300	C11—H11B	0.9700
C2—C3	1.369 (4)	C12—O1	1.207 (3)
C2—H2	0.9300	C12—N1	1.383 (3)
C3—C4	1.378 (4)	C13—C14	1.374 (4)
C3—H3	0.9300	C13—C18	1.379 (4)
C4—C5	1.388 (4)	C13—S1	1.749 (3)
C4—H4	0.9300	C14—C15	1.381 (4)
C5—C6	1.396 (4)	C14—H14	0.9300
C5—C10	1.497 (4)	C15—C16	1.375 (5)
C6—C7	1.503 (4)	C15—H15	0.9300
C7—C8	1.532 (4)	C16—C17	1.381 (5)
C7—H7A	0.9700	C16—C19	1.511 (4)
C7—H7B	0.9700	C17—C18	1.372 (4)
C8—C12	1.504 (4)	C17—H17	0.9300
C8—C9	1.542 (3)	C18—H18	0.9300
C8—H8	0.9800	C19—H19A	0.9600
C9—C10	1.511 (3)	C19—H19B	0.9600
C9—C11	1.528 (4)	C19—H19C	0.9600
C9—H9	0.9800	N1—S1	1.661 (2)
C10—H10A	0.9700	O2—S1	1.428 (2)
C10—H10B	0.9700	O3—S1	1.419 (2)
			
C2—C1—C6	120.1 (3)	N1—C11—C9	104.1 (2)
C2—C1—H1	119.9	N1—C11—H11A	110.9
C6—C1—H1	119.9	C9—C11—H11A	110.9
C3—C2—C1	120.5 (3)	N1—C11—H11B	110.9
C3—C2—H2	119.7	C9—C11—H11B	110.9
C1—C2—H2	119.7	H11A—C11—H11B	108.9
C2—C3—C4	119.4 (3)	O1—C12—N1	124.1 (3)
C2—C3—H3	120.3	O1—C12—C8	127.0 (3)
C4—C3—H3	120.3	N1—C12—C8	108.8 (2)
C3—C4—C5	121.4 (3)	C14—C13—C18	120.4 (3)
C3—C4—H4	119.3	C14—C13—S1	120.8 (2)
C5—C4—H4	119.3	C18—C13—S1	118.7 (2)
C4—C5—C6	118.6 (3)	C13—C14—C15	118.6 (3)
C4—C5—C10	123.6 (3)	C13—C14—H14	120.7
C6—C5—C10	117.8 (2)	C15—C14—H14	120.7
C1—C6—C5	119.8 (3)	C16—C15—C14	122.2 (3)
C1—C6—C7	122.5 (3)	C16—C15—H15	118.9
C5—C6—C7	117.7 (2)	C14—C15—H15	118.9
C6—C7—C8	113.8 (2)	C15—C16—C17	117.7 (3)
C6—C7—H7A	108.8	C15—C16—C19	121.9 (4)
C8—C7—H7A	108.8	C17—C16—C19	120.4 (4)
C6—C7—H7B	108.8	C18—C17—C16	121.4 (3)
C8—C7—H7B	108.8	C18—C17—H17	119.3
H7A—C7—H7B	107.7	C16—C17—H17	119.3
C12—C8—C7	110.1 (2)	C17—C18—C13	119.6 (3)
C12—C8—C9	105.1 (2)	C17—C18—H18	120.2
C7—C8—C9	115.3 (2)	C13—C18—H18	120.2
C12—C8—H8	108.7	C16—C19—H19A	109.5
C7—C8—H8	108.7	C16—C19—H19B	109.5
C9—C8—H8	108.7	H19A—C19—H19B	109.5
C10—C9—C11	113.2 (2)	C16—C19—H19C	109.5
C10—C9—C8	112.0 (2)	H19A—C19—H19C	109.5
C11—C9—C8	105.2 (2)	H19B—C19—H19C	109.5
C10—C9—H9	108.8	C12—N1—C11	112.5 (2)
C11—C9—H9	108.8	C12—N1—S1	123.75 (19)
C8—C9—H9	108.8	C11—N1—S1	123.24 (19)
C5—C10—C9	110.7 (2)	O3—S1—O2	119.80 (15)
C5—C10—H10A	109.5	O3—S1—N1	108.71 (13)
C9—C10—H10A	109.5	O2—S1—N1	104.22 (12)
C5—C10—H10B	109.5	O3—S1—C13	109.13 (14)
C9—C10—H10B	109.5	O2—S1—C13	109.42 (13)
H10A—C10—H10B	108.1	N1—S1—C13	104.41 (12)
			
C6—C1—C2—C3	1.6 (5)	C18—C13—C14—C15	0.5 (4)
C1—C2—C3—C4	−0.4 (5)	S1—C13—C14—C15	−176.2 (2)
C2—C3—C4—C5	−1.4 (5)	C13—C14—C15—C16	−1.6 (5)
C3—C4—C5—C6	2.1 (4)	C14—C15—C16—C17	1.4 (5)
C3—C4—C5—C10	−177.4 (3)	C14—C15—C16—C19	−179.4 (3)
C2—C1—C6—C5	−1.0 (4)	C15—C16—C17—C18	0.0 (5)
C2—C1—C6—C7	176.7 (3)	C19—C16—C17—C18	−179.2 (3)
C4—C5—C6—C1	−0.8 (4)	C16—C17—C18—C13	−1.1 (5)
C10—C5—C6—C1	178.6 (2)	C14—C13—C18—C17	0.8 (5)
C4—C5—C6—C7	−178.6 (2)	S1—C13—C18—C17	177.6 (2)
C10—C5—C6—C7	0.9 (4)	O1—C12—N1—C11	175.1 (3)
C1—C6—C7—C8	142.4 (3)	C8—C12—N1—C11	−4.5 (3)
C5—C6—C7—C8	−39.9 (3)	O1—C12—N1—S1	2.8 (4)
C6—C7—C8—C12	148.4 (2)	C8—C12—N1—S1	−176.76 (18)
C6—C7—C8—C9	29.6 (3)	C9—C11—N1—C12	16.0 (3)
C12—C8—C9—C10	−105.2 (3)	C9—C11—N1—S1	−171.72 (18)
C7—C8—C9—C10	16.3 (3)	C12—N1—S1—O3	−59.7 (3)
C12—C8—C9—C11	18.1 (3)	C11—N1—S1—O3	128.9 (2)
C7—C8—C9—C11	139.6 (2)	C12—N1—S1—O2	171.5 (2)
C4—C5—C10—C9	−133.4 (3)	C11—N1—S1—O2	0.1 (3)
C6—C5—C10—C9	47.2 (3)	C12—N1—S1—C13	56.7 (2)
C11—C9—C10—C5	−172.8 (2)	C11—N1—S1—C13	−114.7 (2)
C8—C9—C10—C5	−54.2 (3)	C14—C13—S1—O3	−10.0 (3)
C10—C9—C11—N1	102.2 (2)	C18—C13—S1—O3	173.2 (2)
C8—C9—C11—N1	−20.3 (3)	C14—C13—S1—O2	122.8 (2)
C7—C8—C12—O1	46.7 (4)	C18—C13—S1—O2	−53.9 (3)
C9—C8—C12—O1	171.6 (3)	C14—C13—S1—N1	−126.1 (2)
C7—C8—C12—N1	−133.7 (2)	C18—C13—S1—N1	57.1 (3)
C9—C8—C12—N1	−8.9 (3)		

### Hydrogen-bond geometry (Å, º)

Cg1 is the centroid of the C1–C6 ring.

**Table:**

*D*—H···*A*	*D*—H	H···*A*	*D*···*A*	*D*—H···*A*
C10—H10*A*···*Cg*1^i^	0.97	2.63	3.555 (3)	159

Symmetry code: (i) −*x*+1, −*y*, −*z*+2.
